# Nerve Injury Evoked Loss of Latexin Expression in Spinal Cord Neurons Contributes to the Development of Neuropathic Pain

**DOI:** 10.1371/journal.pone.0019270

**Published:** 2011-04-29

**Authors:** Hilmar Nils Kühlein, Irmgard Tegeder, Christine Möser, Hee-Young Lim, Annett Häussler, Katharina Spieth, Ingo Jennes, Rolf Marschalek, Tobias Beckhaus, Michael Karas, Markus Fauth, Corina Ehnert, Gerd Geisslinger, Ellen Niederberger

**Affiliations:** 1 *pharmazentrum frankfurt*/ZAFES, Institut für Klinische Pharmakologie, Klinikum der Goethe-Universität Frankfurt am Main, Frankfurt am Main, Germany; 2 Institut für Pharmazeutische Biologie/ZAFES, Goethe-Universität Frankfurt am Main, Frankfurt am Main, Germany; 3 Institut für Pharmazeutische Chemie/ZAFES, Goethe-Universität Frankfurt am Main, Frankfurt am Main, Germany; 4 Institut für Molekulare Biowissenschaften, Goethe-Universität Frankfurt am Main, Frankfurt am Main, Germany; Universidad Federal de Santa Catarina, Brazil

## Abstract

Nerve injury leads to sensitization mechanisms in the peripheral and central nervous system which involve transcriptional and post-transcriptional modifications in sensory nerves. To assess protein regulations in the spinal cord after injury of the sciatic nerve in the Spared Nerve Injury model (SNI) we performed a proteomic analysis using 2D-difference gel electrophoresis (DIGE) technology. Among approximately 2300 protein spots separated on each gel we detected 55 significantly regulated proteins after SNI whereof 41 were successfully identified by MALDI-TOF MS. Out of the proteins which were regulated in the DIGE analyses after SNI we focused on the carboxypeptidase A inhibitor latexin because protease dysfunctions contribute to the development of neuropathic pain. Latexin protein expression was reduced after SNI which could be confirmed by Western Blot analysis, quantitative RT-PCR and in-situ hybridisation. The decrease of latexin was associated with an increase of the activity of carboxypeptidase A indicating that the balance between latexin and carboxypeptidase A was impaired in the spinal cord after peripheral nerve injury due to a loss of latexin expression in spinal cord neurons. This may contribute to the development of cold allodynia because normalization of neuronal latexin expression in the spinal cord by AAV-mediated latexin transduction or administration of a small molecule carboxypeptidase A inhibitor significantly reduced acetone-evoked nociceptive behavior after SNI. Our results show the usefulness of proteomics as a screening tool to identify novel mechanisms of nerve injury evoked hypernociception and suggest that carboxypeptidase A inhibition might be useful to reduce cold allodynia.

## Introduction

Injury to peripheral or central nerves may result in the development of neuropathic pain [1]. Despite recent advances in the understanding of the pathophysiology of this disease it is still unclear why adaptive processes elicited by the injury allow for a recovery of stability and normal neuronal excitability in the majority of patients but fail in others [1]–[4]. Because injured neurons adapt protein degradation and de novo synthesis to prepare for the reorganization of signaling and synaptic functions, proteome analyses from afflicted sites are likely to further unravel the mechanisms and unfavorable regulations which challenge the recovery of balance [5]. In the present study we used the Spared Nerve Injury model of neuropathic pain [6] to screen for proteomic manifestations in the spinal cord. Based on the hypothesis that neuropathic pain may arise from a dys-balance of activator/inhibitor or agonist/antagonist protein pairs we focused on endogenous enzyme inhibitors. The analysis identified latexin as a potential functionally relevant downregulated candidate. Latexin is the only known endogenous inhibitor of the C-terminal exopeptidase, carboxypeptidase A, which preferentially cleaves off C-terminal hydrophobic L-amino acids that have aromatic or branched side chains [7], [8]. Carboxypeptidase A isoforms contribute to the processing of opioid peptides, neurotensin, corticotropin, angiotensin and other neuropeptides [9]–[12]. It is therefore likely that a dys-balance between carboxypeptidase A and its inhibitor, latexin may affect neuropeptide signaling in the spinal cord. Particularly, endogenous opioid peptides are essential mediators in the endogenous defense against pain and dysfunctions in endorphin or enkephalin degradation may aggravate hyperexcitability of nociceptive synapses [13]. Latexin is expressed in subsets of neurons of the peripheral and central nervous system including cortical neurons in the SII somatosensory cortex [14], [15]. Recent studies suggest that latexin deficient mice have a phenotype in some nociceptive tests but not in others [15]. Based on our hypothesis and based on previous evidence we analyzed here the regulation and function of latexin in the spinal cord and dorsal root ganglia in the context of neuropathic pain.

## Methods

### Animals and treatments

#### Ethics Statement

In all animal experiments the ethic guidelines for investigations in conscious animals were obeyed and the procedures were approved by the local Ethics Committee for Animal Research (Regierungspräsidium Darmstadt, Germany).

For the proteomic analysis male Sprague Dawley rats (Charles River, Sulzbach, Germany) weighing 260–300 g were used. They were housed in groups of five in standard cages and maintained in climate- and light-controlled rooms (22±0.5°C, 12/12 h dark/light cycle) with free access to food and water. To assess the effect of latexin transduction or carboxypeptidase inhibitor on the neuropathic pain behavior we used C57BL/6 mice to reduce the amount of viruses and drug which would have been needed to modulate latexin or carboxypeptidase activity in the spinal cord of rats, respectively.

### Nerve injury

The spared nerve injury (SNI) model was used as described previously [6], [16]. Briefly, animals were anesthetized with isoflurane, and the tibial and common peroneal branches of the sciatic nerve were ligated and sectioned distally, whereas the sural nerve was left intact. For sham surgery the sciatic nerve was exposed but not touched. Sham operated and naïve animals were used as controls. Animals were sacrificed at the indicated time points after surgery and the L4/L5 DRGs (ipsi- and contralateral) and the lumbar spinal cord (L4/5) were dissected for further analysis. The spinal cord was then further prepared to separate the ipsi- and contralateral dorsal and ventral horns. Therefore, lumbar spinal cords were placed under a microscope and the sections were prepared by a micro scalpel. For proteomic studies nine animals were analyzed in each group. For RNA analysis and *in situ* hybridisation three and six animals, respectively, have been used in each group.

### Preparation of protein extracts

Protein extracts from DRGs and lumbar dorsal horn tissue were prepared using a lysis buffer containing 8 M urea, 4% CHAPS, 30 mM Tris, 1 mM PMSF pH 8.5. After removal of cellular debris extracts were ultracentrifuged at 40,000 rpm for 1 h (4°C) and the supernatant was stored at −80°C until analysis. Protein concentrations were determined by the Bradford protein assay.

### 2-D DIGE Analysis

For DIGE analysis 9 rats/group have been used and 3 gels were run/experiment. Individual protein samples (50 µg) were minimally labelled with the fluorescent dyes Cy2 (SNI dorsal horn ipsilateral, 7 days after surgery), Cy3 (internal standard), or Cy5 (sham operated dorsal horn ipsilateral, 7 days after surgery) (400 pmol dye each, GE Healthcare) according to manufacturer's instructions. As internal standard a protein mixture consisting of control and SNI samples was used. Fluorescence labelling was carried out for 30 min in the dark, and the reaction was stopped by the addition of 1 µl 10 mM L-lysine for 10 min on ice in the dark. Labelled samples were applied to rehydrated 18 cm non-linear pH 3–10 IPG strips (Immobiline DryStrips, GE Healthcare) by anodic cup loading and IEF was performed for a total of 56000 Vh on an IPGphor system (GE Healthcare). After IEF, the strips were equilibrated at room temperature for 15 min in 50 mM Tris-HCl, 6 M urea, 30% (v/v) glycerol, 2% SDS (w/v) and 0.002% (w/v) bromphenol blue containing 1% (w/v) DTT and then, for 15 min in the same buffer without DTT, but with the addition of 4.8% (w/v) iodoacetamide. Proteins were further separated according to their molecular weight on SDS-PAGE (12.5%) using an Ettan DALT*six* system (GE Healthcare). Following electrophoresis, gels were fixed and then scanned at appropriate wavelengths for Cy2, Cy3, and Cy5 fluorescence using a Typhoon 9400 (GE Healthcare) scanner. Protein expression was quantified using DeCyder Batch Processor and Biological Variation Analysis (BVA) software V5.02.

### In-gel digestion and MALDI-TOF-MS

Protein spots showing an SNI-evoked change of expression of at least 40% were picked using an Ettan Spot Picker. Cut out gel pieces were subjected to in-gel digestion protocols [17] which were adapted for use on a Microlab Star digestion robot (Bonaduz, Switzerland) [18]. Briefly, samples were reduced, alkylated and subsequently digested using bovine trypsin (Roche, Mannheim, Germany). The digestion mixture was dissolved in 5 µl of 50% acetonitrile/1% trifluoroacetic acid (TFA). The samples (0.5 µl) and 0.5 µl of matrix (2 mg/ml 1-cyano-4- hydroxycinnamic acid in 50% acetonitrile/0.5% TFA) were consecutively spotted and dried in ambient air. MALDI-TOF mass spectra were recorded on an Ultraflex TOF/TOF (Bruker Daltonics, Bremen) using a nitrogen laser (λ = 337 nm, repetition rate = 25 Hz) for desorption and ionisation with an acquisition mass range from 700 to 5000 m/z. The low mass gate was set to 650 m/z. Spectra were externally calibrated with Sequazyme Peptide Mass Standards Kit (Applied Biosystems, Darmstadt). Between 1000 and 2000 single scans were accumulated for each mass spectrum. Monoisotopic peaks were labeled using flex analysis V2.2. Proteins were identified using MASCOT 2.0 (Matrixscience) installed on a local server using the current National Centre for Biotechnology Information (NCBI) database (NCBInr 20050721 (2693904 sequences; 923764693 residues, Timestamp: 25 Jul 2005 at 14∶15∶12 GMT) without species restriction. For peptide-mass fingerprinting, the most intense peaks were submitted using a search with the following parameters: Enzyme: Trypsin; Fixed modification: Carbamidomethyl (C), variable modifications: oxidation (M), mass values: monoisotopic, protein mass: unrestricted, peptide mass tolerance: 50 ppm, peptide charge state: 1+, Max missed cleavages:1). Proteins with a score of 77 or higher were considered significant (p<0.05).

### Western Blot analysis

Protein lysates (30 µg) were separated by 12% SDS–PAGE. Proteins were then transferred onto nitrocellulose membranes by wet blotting. To confirm equal loading all blots were stained with Ponceau red solution. Membranes were blocked for 60 minutes at room temperature in Odyssey blocking reagent (LI-COR Biosciences) diluted 1∶2 in PBS. Then the blots were incubated overnight at 4°C with primary antibody against carboxypeptidase A (37 kDa) (1∶400, Biogenesis, Poole, UK) or latexin (28 kDa) (1∶100, Santa Cruz, Heidelberg, Germany) in blocking buffer. After washing three times with 0.3% Tween 20 in PBS, the blots were incubated for 60 min with an IRDye800- or IRDye700-conjugated secondary antibody (1∶10000 in blocking buffer). After rinsing in 0.3% Tween 20 in PBS, protein-antibody complexes were detected with the Odyssey Infrared Imaging System (Licor, Bad Homburg, Germany). ERK-2 (42 kDa) (1∶2500, Santa Cruz, Heidelberg, Germany) was used as loading control.

### Carboxypeptidase activity assay

CPA activity in preparations of the lumbar spinal cord has been determined as described previously [19]–[21] with slight modifications. As CPA substrate we used N-Acetyl-L-phenylalanyl-L-3-thiaphenylalanine (Peptides International, Louisville, USA) which is hydrolysed by active CPA. The released thiophenole reacts to a yellow dye by addition of Ellmans reagent. The absorbance of the product was measured photometrically at 410 nm (Spectrafluor Plus, Tecan, Crailsheim, Germany).

### Quantitative RT-PCR

Total RNA was extracted from tissues and cells as described previously [22]. mRNA expression was analysed using a One-step RT-PCR Kit (Qiagen, Hilden, Germany). Primers used for RT-PCR analysis are shown in Table 1.

**Table 1:** Primers used for RT-PCR analysis.

Gene	Accession number	Abbrevation	Amplification product (bp)	Primer sequence
Annexin A4	NM_024155	*Anxa4*	248	FW 5′-GCAGAGATTGACATGCTGGA-3′RV 5′-CTGGAGGCGTTTTAATTGGA -3′
Latexin	NM_031655	*Lxn*	420	FW 5′-GGAAGAGGCCACAAGTACCA-3′RV 5′-GTCGTGGAGTAGGACGGTGT -3′
Peptidylprolyl isomerase A	NM_017101	*Ppia*	248	FW 5′-AGCACTGGGGAGAAAGGATT -3′RV 5′- AGCCACTCAGTCTTGGCAGT-3′
Prohibitin	M61219	*Phb*	187	FW 5′- GGCAGCCTGAGTAGACCTTG-3′RV 5′- TCACGGTTAAGAGGGAATGG-3
Cytochrome c oxidase	XM_578078	*CCO*	235	FW 5′- GCTGGCAGAACTACCTGGAC-3′RV 5′- GAGGTCCCCCTTTTCTATCG-3′
Ubiquitin carboxy-terminal hydrolase	D10699	*UCH*	189	FW 5′- CTAGGGCTGGAGGAGGAGAT-3′RV 5′-CCCAATGGTACCACAGGAGT-3′
Pyruvate dehydrogenase	U10357	*PD*	149	FW 5′- AGGAAGTCAATGCCACCAAC-3′RV 5′- TTTTGATGGGAGGGAGAGTG-3
Serine/threonine-specific protein kinase	AF068261	*STK*	200	FW 5′- CAAGAAGGTCAGGCGAGTTC-3′RV 5′-ACTGTATTTGCTCGGGGATG-3′
Myelin basic protein	K00512	*MBP*	176	FW 5′- GCACCCTGACTGGCTAAAAC-3′RV 5′- CTCGCCGTGAAAAGAAAGTC-3′
Glutathione-S-transferase, mu 5	NM_172038	*GSTM5*	210	FW 5′- GCACAACATGTGTGGTGACA -3′RV 5′- AGCTTTTCTCCTGCAAACCA -3′
NADH dehydrogenase (ubiquinone) Fe-S protein 3	XM_215776	*NADH DH*	243	FW 5′- AGCAGTGGATGTCCCAACTC-3′RV 5′- TGTCCCTCGAAGCCATAATC-3′
Phosphoglycerate mutase	S63233	*PGM*	198	FW 5′- CTCAGGGCAAGGTGAAGAAG-3RV 5′- GGTGCCGGGGATAAAATACT-3′
Beta-actin	NM_031144	*Actb*	300	FW 5′-CAGCGGAACCGCTCATTGATGG-3′,RV 5′-TCACCCACACTGTGCCCAACGA-3′

Reverse transcription reaction was performed with specific primers at 50°C for 30 min. PCR amplification was started by enzyme activation at 95°C for 10 min. The samples were then denatured at 95°C for 1 min, annealed at 58°C 1 min and extended at 72°C for 1 min in 35 repetitive cycles. After a final extension at 72°C for 10 min the PCR was stopped and the samples were separated by 1% agarose gel electrophoresis. The amplified cDNA bands were detected by ethidium bromide staining.

For Taqman analysis cDNA synthesis was performed with random hexamers using the Superscript III kit (Invitrogen GmbH, Karlsruhe, Germany). The expression levels of latexin were determined by SYBR Green labeling (Abgene Limited, Epsom, United Kingdom) with an ABI Prism 7500 Sequence Detection System (Applied Biosystems, Austin, USA). The variability of mRNA input was normalized to the amount of 18SRNA.

### *In situ* hybridization

A cDNA was reverse transcribed from rat spinal cord mRNA with random primers. The primers 5′-AAATCCCACCCACCCACTAC-3′ and 5′-ATTGTGGGTGCCACAGAACT-3′ were used to synthesize a fragment corresponding to nucleotides 8–616 of latexin mRNA (NM_031655) by PCR. Agarose gel electrophoresis presented a single band with the expected size. The fragment was cloned into the pCR4-TOPO plasmid vector (Invitrogen, Karlsruhe, Germany) and transformed into *E. coli*. Isolated plasmid was linearized with *Not*I and *Pme*I and digoxigenin-labeled sense and antisense probes were produced by reverse transcription using T3 or T7 RNA polymerases, respectively.

Lumbar spinal cord as well as dorsal root ganglia (DRG, L3–L5) were dissected and immediately frozen in tissue freezing medium on dry ice. Cryostat sections were cut at a thickness of 16 µm and fixed in 4% paraformaldehyde in 0.1 M PBS (pH 7.4) for 10 min. After rinsing in PBS, samples were acetylated with acetic anhydride in 0.1 M triethanolamine for 10 min, pre-hybridized in hybridization buffer (50% formamide, 5× SSC (diluted from a 20×SSC stock solution: 3 M NaCL, 0.3 M Na-Citrat, pH 7.0), 5× Denhardt's solution, 500 µg/ml herring sperm DNA, 250 µg/ml yeast tRNA) at 70°C for 2 h, and incubated with latexin sense or antisense probes in hybridization buffer (300 ng/ml) at 70°C overnight. After hybridization, the slides were washed twice in 0.2× SSC and TBS (0.1 M Tris-HCl, 0.15 M NaCl, pH 7.5) at 70°C, incubated for 1 h in blocking buffer (0.12 M maleic acid, 0.15 M NaCl, pH 7.5; 1% Blocking Reagent; Roche Diagnostics, Mannheim, Germany) followed by incubation of alkaline phosphatase-conjugated anti-digoxigenin antibody (1∶1000, Roche Diagnostics) in blocking buffer at 4°C overnight. Sections were then washed in TBS and equilibrated in alkaline buffer (0.1 M Tris-HCl, 0.1 M NaCl, 0.05 M MgCl_2_, pH 9.5, 2 mM levamisole). A colorimetric reaction using BM Purple AP substrate (Roche Diagnostics) was performed at room temperature for 1–2 h. Images were obtained using an Eclipse E600 microscope equipped with a Kappa DX 20 H camera and Kappa ImageBase software. BM Purple-stained latexin mRNA was visualized using brightfield illumination.

### Preparation of latexin-containing adenovirus

Full length latexin cDNA was obtained by using total RNA from mouse spinal cord using specific mouse latexin primers, carrying *BamHI* and *Nhe*I restriction sites (RV with *BamHI* restriction site (underlined): 5′-CGT CTT GGA TCC TCC GCC TGC CCT TC-3′; FW with *Nhe*I restriction site (underlined): 5′-TCT CCG CTA GCA TGG AAA TCC CAC CCA CCC AC-3′) (Biospring, Frankfurt, Germany). The 665 bp PCR product encoding latexin was inserted into the multiple cloning site of a neuron-specific adenovirus associated vector (AAV2) in front of green fluorescence protein (EGFP) as a fusion product. In this vector latexin-EGFP expression is controlled by the human synapsin 1 promoter to ensure neuron specificity [23], [24]. The vector was amplified in *E.coli* DH5α and selected positive recombinants were confirmed by sequencing analysis by AGOWA, Berlin, Germany. Recombinant adenoviruses were produced in 293A HEK cells (Microbix, Canada) by transient co-transduction of AAV2 with two helper plasmids containing rep2 and caps1 genes and adenovirus viral-associated (VA) RNA polymerase I and II genes. A standard calcium-phosphate method was used for transduction and the cells were maintained in Dulbecco's modified Eagle's medium (Invitrogen) supplemented with 10% fetal bovine serum (Hyclone), 1% glutamate, and 1 mM sodium pyruvate (Invitrogen) in a 37°C incubator with 5% CO_2_. Six hours after transduction, the medium was replaced by fresh pre-warmed DMEM culture medium containing 2% FCS. Two-three days post-transduction, the efficiency of viral infection was determined by EGFP fluorescence microscopy (Zeiss Axio-Imager, Zeiss, Germany) and FACS analysis. Cells were lysed by three freezing/thawing cycles and adenovirus stocks were stored at −80°C till use. Latexin transduction of human neuroblastoma cell cultures (SH-SY5Y) was analysed by RT PCR 3 days after transduction with AAV2-Syn-Latexin-EGFP.

### Intrathecal injection of adenovirus in mice

Mice were anesthetized with a mixture of fentanyl (2.1 mg/kg)/midazolam (21 mg/kg) to minimize respiratory thoracic movements during surgery. After blunt removal of back muscles and ligaments we performed a minimal hemi-laminectomy above the L4–5 lumbar spinal cord. Using a Nanoliter Injector (World Precision Instruments (WPI), Berlin, Germany) equipped with a glass capillary we injected a total of 372 nl of AAV2-hSyn-Latexin-EGFP solution (10^11^–10^12^ VP/ml) carrying the latexin cDNA or empty AAV2 carrying an identical expression cassette without transgene (control) into the ventral and dorsal horns at 3 adjacent sites of the L4–5 lumbar spinal cord ipsilateral to the nerve lesion. The injection was performed 7 days before SNI surgery. Nociceptive behavior was assessed before virus injection and 7 days after virus injection before nerve injury to determine the baseline and then at days 3, 7, 14, 21 and 28 after nerve injury. 13–14 animals were used in each group.

### Oral treatment with carboxypeptidase inhibitor

To inhibit carboxypeptidase activity in mice we used the small molecule DL-benzylsuccinic acid (Sigma) which has been described as potent carboxypeptidase inhibitor [25]. The drug was dissolved at a concentration of 1 mg/ml in tap H_2_O and then administered at a dose of 200 mg/kg body weight/day with the drinking water. Treatment started 3 days before SNI surgery. Baselines for nociceptive behavior were determined before the first drug administration and then at day 3 after drug treatment before surgery. The nociceptive behavior after SNI was assessed on days 3, 7 and 14. Six mice have been used in each group.

### Analysis of nociception

Behavioral tests were performed in a silent room with constant temperature (21–23°C) during daylight after adaptation of the animals to the test cages with a metal grid bottom. The experimenter was blinded to treatment of the animals. All animals have been used to assess both parameters. The experiments were performed in the same order in each measurement.

#### Mechanical hyperalgesia

Paw withdrawal latency to mechanical stimulation was assessed with an automated testing device consisting of a steel rod that was pushed against the plantar surface of the paw with increasing force until the paw was withdrawn (Dynamic Plantar Aesthesiometer, Ugo Basile, Varese, Italy). The maximum force was set at 5 g and the ramp speed was 0.5 g/s.

#### Cold allodynia

To assess cold allodynia in the SNI-model a drop of acetone was applied with help of a 1 ml syringe onto the plantar side of the hindpaw ipsilateral to the nerve lesion. The time the mice spent lifting, shaking or licking the acetone treated paw was recorded with a stop watch during an observation period of 2 min starting right after acetone application.

### Immunofluorescence

Mice were intracardially perfused with 0.9% saline followed by 4% paraformaldehyde in 0.1 M phosphate-buffered saline (PBS, pH 7.4) under deep ketamine/xylazine anesthesia. The lumbar spinal cord was dissected, post-fixed in the same fixative for 2.5 h and cryoprotected in 30% sucrose overnight. Tissues were frozen in tissue freezing medium on dry ice and cryostat-sectioned at a thickness of 14 µm. Slices were permeabilized for 15 min with PBS containing 0.1% Triton-X 100. The sections were then blocked in 3% BSA in PBS for 1 h to reduce nonspecific binding and incubated overnight at 4°C with the primary antibody against neuronal nuclear protein (NeuN) to confirm neuron-specific infection with the AAV2-hSyn-Latexin-EGFP. Species specific secondary antibody labelled with Cy3 was used for microscopic detection. Adenovirus infection was detected by the EGFP signal. Slides were mounted with SlowFade Light Antifade mounting media according to manufacturer's protocol (Molecular Probes, Leiden, The Netherlands) and then analysed using a Zeiss AxioImager fluorescence microscope.

### Data analysis and statistics

Statistical evaluation was done with SPSS 12.0 for Windows. Data are presented as means ± SEM. For comparison of Western Blot and TAqman PCR analysis we applied unpaired Students t-test. For statistical comparisons of behavioral data we submitted time course data to an analysis of variance (ANOVA) for repeated measurements and calculated the area under the time curve (AUC) employing the linear trapezoidal rule. AUCs were subsequently compared with unpaired Student's t-tests (AAV2-hSyn-Latexin-EGFP versus control AAV2). For all tests, a probability value P<0.05 was considered as statistically significant.

## Results

### Differential protein expression following spared nerve injury

**Figure 1:**
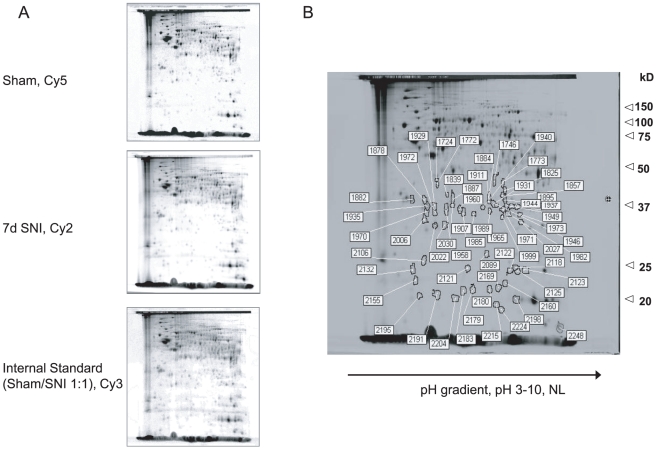
DIGE analysis. (A) Representative 2D-gels of the lumbar spinal cord of sham-operated rats, 7 days after nerve injury and internal control (Sham/SNI 1∶1). (B) Overlay of the 3 images indicating regulated protein spots.

The proteome of spinal cord lumbar dorsal horn samples from SNI and sham operated rats was analyzed by 2D-DIGE. An average of 2300 protein spots/gel distributed in a range of p*I* 4.0–9.0 and MW between 20–150 kD were detected. Differential expression was considered at >40% difference of the spot intensity. We found 55 significantly regulated proteins from which one spot was up- and 54 were down-regulated after SNI. Fig. 1A shows representative 2D-gels of dorsal horn protein extracts of sham-operated rats, 7 days after SNI and the internal control (Sham/SNI 1∶1), respectively. Proteins that were at least 40% regulated 7 days after nerve injury are indicated in the overlay image of the 3 gels (Fig. 1B). For comparison, a regulation of only 20% spot intensity revealed 91 down-regulated and 18 up-regulated proteins (data not shown). The 55 regulated spots were picked and identified by peptide-mass fingerprinting using MALDI-TOF-MS with subsequent Mascot database search. With this procedure we identified 41 proteins (Table S1) which are mostly involved in energy metabolism, cellular structure, signal transduction and DNA binding.

### Analysis of gene expression of altered proteins by RT-PCR

Gene expression analysis of 12 selected proteins which are potentially involved in nociceptive transmission was performed by RT-PCR to further assess the regulation at the transcriptional level. In concordance with the protein level, we found that mRNAs were down regulated for prohibitin (PHB), Annexin A4 (Anxa4), pyruvate dehydrogenase (PD), ubiquitin carboxyterminal hydrolase (UCH), and latexin (Lxn) 7 days after nerve injury. Interestingly, phosphoglycerate mutase (PGM) mRNA was significantly upregulated while protein levels were reduced in 2D-PAGE. The mRNA of peptidylprolyl isomerase A (Ppia), glutathione S-transferase, mu 5 (GSTM5), myelin basic protein (MBP), serine/threonine specific kinase (STK), NADH dehydrogenase (NADH DH) and cytochrome C oxidase (CCO) showed no significant differences between the ipsi- and the contralateral side at the transcriptional level indicating that the observed down regulation in the DIGE-analysis was due to translational or post-translational modifications or changes of the subcellular localization. We additionally determined mRNA levels of these candidate regulated proteins in the L4/5 DRGs using the same primers. The results were similar to the spinal cord: PHB, PD, GSTM5, and CCO were significantly down regulated 7 d after SNI while Anxa4, Lxn, NADH DH, STK, PGM, UCH MBP and Ppia remained unaltered (Table 2).

**Table 2:** mRNA expression in the spinal cord and the DRGs.

	Spinal Cord		DRGs	
	DH contra [%] ± SEM	DH ipsi [%] ± SEM	DRG contra [%] ± SEM	DRG ipsi [%] ± SEM
Prohibitin	**100.0±1.78**	60.5±2.56***	**100.0±3.27**	71.3±4.61**
Pyruvate dehydrogenase	**100.0±2.39**	63.7±7.48**	**100.0±2.09**	82.8±4.01 *
Annexin A4	**100.0±1.86**	83.1±2.95**	100.0±1.50	78.2±10.14
Latexin	**100.0±1.20**	79.7±7.20*	100.0±3.71	87.2±4.43
Ubiquitin carboxy-terminal hydrolase	**100.0±15.46**	43.2±5.51*	100.0±2.94	96.9±8.19
Phosphoglycerate mutase	**100.0±1.91**	106.6±1.82*	100.0±4.34	94.5±3.91
Cytochrome c oxidase	100.0±3.85	114.7±10.84	**100.0±1.19**	91.9±1.87*
Glutathione-S-transferase. Mu 5	100.0±8.26	102.8±6.92	**100.0±2.90**	79.3±5.18*
Myelin basic protein	100.0±0.52	103.2±4.98	100.0±6.78	97.1±1.31
NADH dehydrogenase (ubiquinone) Fe-S protein 3	100.0±1.92	109.6±8.86	100.0±5.32	87.7±9.77
Serine/threonine-specific protein kinase	100.0±1.02	107.5±4.39	100.0±8.65	90.0±10.40
Peptidylprolyl isomerase A	100.0±3.92	92.3±2.05	100.0±1.75	102.9±2.86

mRNA expression in the ipsi- and contralateral dorsal horn (DH) of the spinal cord and the DRGs, respectively. Significantly regulated mRNA levels are indicated by bold numbers (*P<0.05, **P<0.01; ***P<0.001).

### Regulation and localisation of the carboxypeptidase A inhibitor latexin

**Figure 2:**
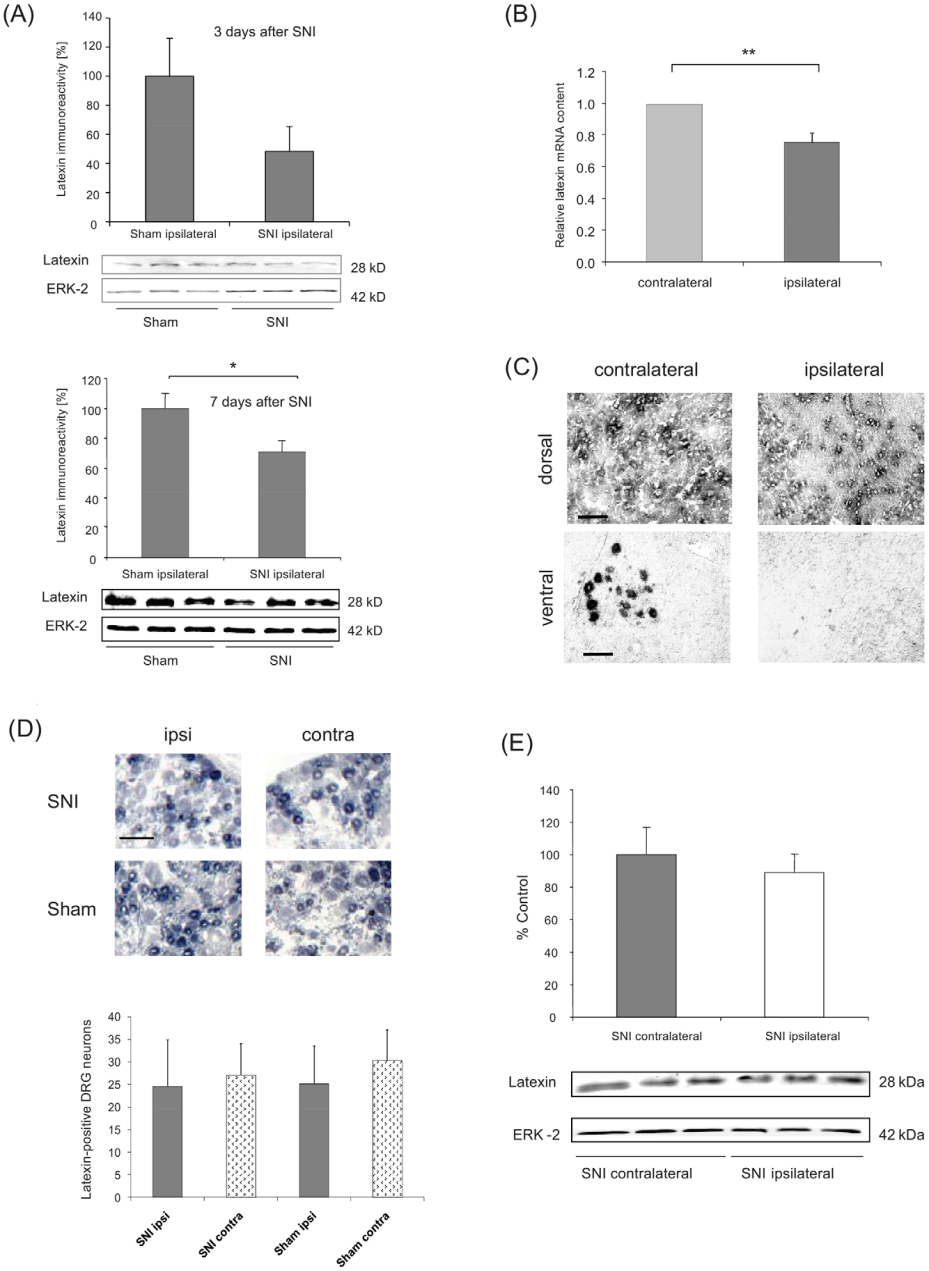
Latexin regulation in the spinal cord and the dorsal root ganglia (DRGs). (A) Western Blot showing latexin protein regulation in the ipsilateral dorsal horn of the spinal cord in rats three (P = 0.25) and seven days after nerve injury in the Spared Nerve Injury model (SNI). The diagram depicts the result from the densitometric analysis of the blots (n = 3 animals/group) (B) mRNA expression in the ipsi- and contralateral spinal cord after SNI as investigated by quantitative RT-PCR (Taqman) (C) Latexin mRNA in the dorsal and ventral spinal cord ipsi- and contralateral to the nerve lesion analysed by *in situ* hybridisation. (D) *In situ* hybridisation of latexin in ipsi- and contralateral DRGs in SNI- and sham-operated rats. The diagram shows the mean counts of latexin-positive neurons in the respective DRGs. (E) Western Blot showing latexin protein regulation in the ipsi- and contralateral DRGs in SNI-operated rats. The diagram depicts the result from the densitometric analysis of the blots. Data are shown as mean ± SEM, ^*^ *P*<0.05, Scale bar: 100 µm.

Since latexin has been described for its expression in pain-relevant tissues in the peripheral and central nervous system [7], [26], [27] and has been implicated in pain-signalling pathways [14], [15] we focused on the functional analysis of latexin. Western Blot analysis revealed a significant downregulation of latexin protein levels in the dorsal horn of the spinal cord ipsilateral to the nerve lesion in comparison to sham operated control rats 7 days after surgery thus confirming the data obtained from the DIGE analysis (Figure 2A). Quantitative Taqman RT-PCR showed a significant decrease in latexin mRNA after SNI in the spinal cord (Figure 2B). *In situ* hybridization demonstrated latexin mRNA expression in dorsal horn and motor neurons in the spinal cord and in sensory neurons in the DRGs (Figure 2C and 2D). Seven days after SNI surgery we observed a decrease in latexin mRNA in the spinal dorsal and ventral horns ipsilateral to the nerve lesion (Figure 2C). The mRNA expression of latexin in the DRGs did not change after SNI (Figure 2D) and Western Blot analysis also did not reveal differences in latexin protein levels in the DRGs (Figure 2E).

### Carboxypeptidase expression and activity after SNI

**Figure 3:**
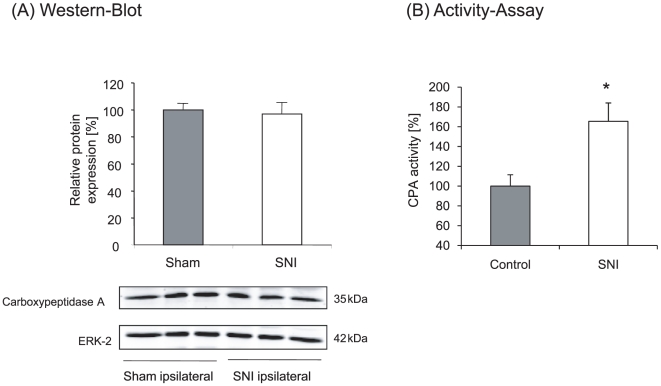
Carboxypeptidase A (CPA) expression and activity in the spinal cord. (A) Western Blot analysis of CPA protein levels and (B) CPA activity in the ipsilateral spinal cord of sham and SNI operated animals. Data are shown as mean ± SEM, *P<0.05 (n = 5–6/group). For better comparison, controls are set as 100%.

Since latexin is an endogenous inhibitor of carboxypeptidase A in mammals a modulation of latexin expression may affect carboxypeptidase activity. Western Blot analysis showed no differences in the protein levels of carboxypeptidase A in the ipsilateral dorsal horn of the spinal cord after SNI (Figure 3A). However, activity assays revealed an increase of CPA-activity after SNI which is in line with the decreased expression of its inhibitor latexin (Figure 3B).

### Neuropathic pain after adenovirus-mediated prevention of latexin downregulation

**Figure 4:**
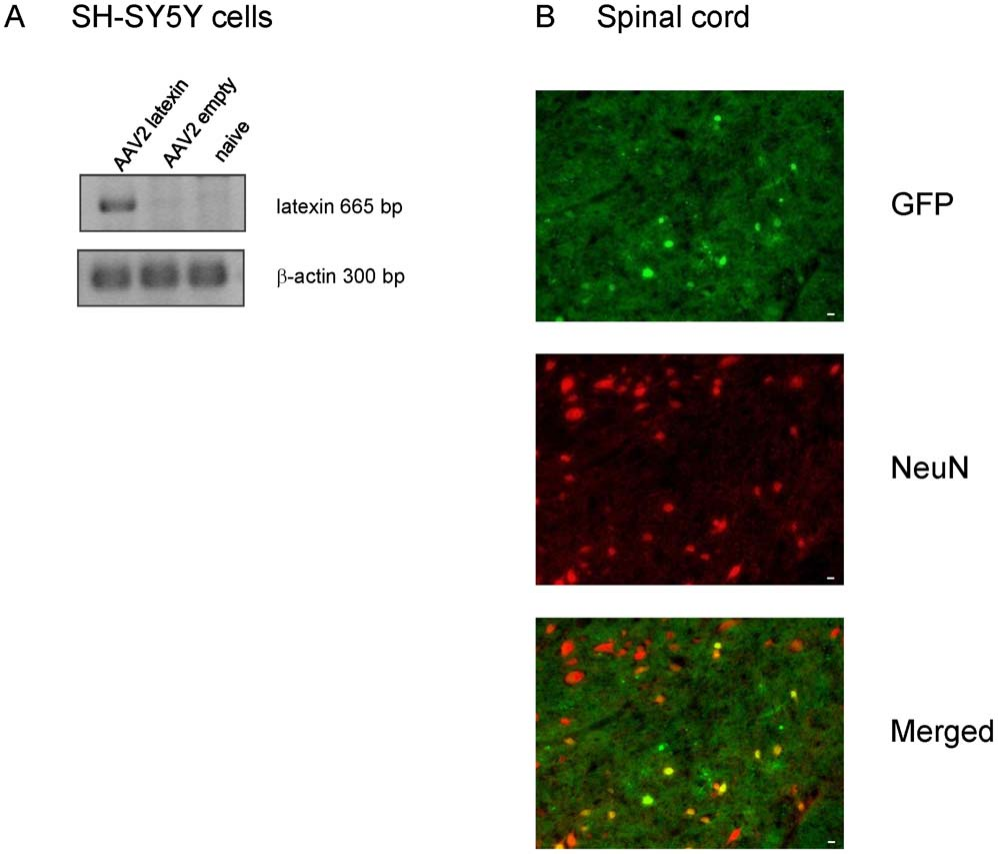
AAV-mediated overexpression of latexin. (A) RT PCR of latexin in SH-SY5Y neuroblastoma cells (naive or transduced with adenovirus with and without latexin) and (B) Spinal cord slices of mice 7 days after injection of AAV2 latexin virus. Upper panel: EGFP staining, middle; NeuN immunofluorescence, Lower panel Merged; Scale bar 10 µm.

We next assessed whether the nerve injury-evoked latexin loss in the spinal cord promoted the development of neuropathic pain. To prevent the loss of latexin we used an AAV-mediated transduction approach to enhance and restore latexin expression in the spinal cord ipsilateral to the nerve lesion. We used mice to facilitate surgery and transduction in these experiments. The downregulation of latexin after SNI was equivalent in mice and rats (data not shown). Recombinant rAAV2 (rAAV2-hSyn-latexin-EGFP) mediated enhancement of latexin expression was confirmed in human neuroblastoma cells (SH-SY5Y) (Figure 4A). In vivo, we assessed successful latexin transduction in the spinal cord by microscopic analysis of EGFP signals. Co-immunostainings with marker antibodies for neurons confirmed the intended neuron specificity (Figure 4B). We found positive neurons in an area of few millimetres surrounding the injection sites.

**Figure 5:**
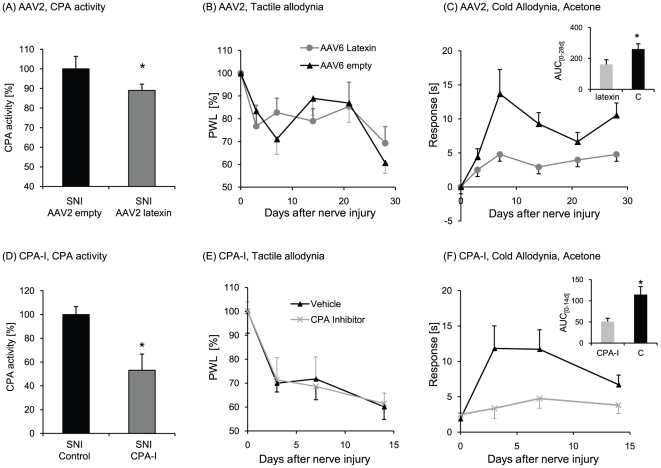
Nociceptive behavior after CPA-inhibition either by AAV-mediated latexin rescue or the CPA inhibitor D, L-benzylsuccinic acid. (A) CPA activity in the spinal cord of SNI treated mice injected either with adenoassociated virus backbone (empty AAV2) or AAV2-latexin. (n = 5–6/group). CPA activity of controls is set at 100%. (B) Mechanical hyperalgesia assessed with a dynamic von Frey apparatus and (C) cold allodynia assessed in the acetone test in AAV2 treated mice. (n = 13–14 animals in each group), ▴ empty AAV2, • AAV2 latexin. (D) CPA activity in the spinal cord of SNI treated mice with or without oral administration of the CPA inhibitor racemate (CPA-I) D, L-benzylsuccinic acid. (n = 5/group). Controls are set at 100%. (E) and (F) mechanical hyperalgesia and cold allodynia in control and D, L-benzylsuccinic acid treated mice, respectively. (n = 6 animals in each group), ▴ controls, X CPA-Inhibitor. Data are means ± SEM, *P<0.05.

In experiments where we prevented the SNI evoked latexin decrease by rAAV2-mediated neuron-specific latexin transduction in the ipsilateral spinal cord carboxypeptidase A activity decreased. This confirmed the reciprocal relationship between latexin expression and carboxypeptidase A activity. The neuropathic pain-like behavior was assessed over a period of 28 d by monitoring cold allodynia and mechanical hyperalgesia. Mechanical nociception was identical in mice tranduced with rAAV2-hSyn-latexin-EGFP or control virus which contained an identical expression cassette without transgene. However, cold allodynia was significantly reduced after treatment with rAAV2-hSyn-latexin-EGFP (repeated measure ANOVA p<0.001; t-test of AUC_[0–28d]_ p<0.05) (Figure 5 A–C) indicating that normalization of latexin expression was able to prevent the development of cold-induced hypernociception.

Mice treated orally with the carboxypeptidase inhibitor D,L-benzylsuccinic acid showed a significant reduction in carboxypeptidase activity in the spinal cord. This was associated with a significant reduction of cold allodynia but not mechanical hyperalgesia. Hence, the effects of CPA inhibition with a small molecule inhibitor resembled those of latexin transduction (Figure 5 D–F). The result suggests that carboxypeptidase A contributes to the development of cold allodynia, but not mechanical hyperalgesia.

## Discussion

Neuropathic pain is characterized by hypersensitivity in the peripheral and central nervous system and urgently requires specific and effective treatment. This is often constricted since currently available drugs are ineffective or associated with severe dose-limiting side effects. A better knowledge of the molecular mechanisms of neuropathic pain which involve a number of changes in the protein expression pattern in neuronal tissues might facilitate the development of novel therapies. In the present study, proteomics revealed latexin as a candidate protein involved in nerve injury evoked hypernociception. We found abundant expression of latexin in dorsal and ventral horn neurons in the spinal cord where it functions as carboxypeptidase inhibitor. Its strong downregulation after nerve injury resulted in increased CPA activity. Counter-regulation of this process by AAV-mediated latexin cDNA delivery or administration of a carboxypeptidase inhibitor reduced neuropathic pain-like behavior suggesting that the latexin/carboxypeptidase A system may contribute to the manifestation of cold allodynia in the SNI model.

The proteomic 2D-DIGE approach used in the present study revealed the regulation of several proteins in the dorsal horn of the spinal cord following a peripheral lesion of the peroneal and tibial branches of the sciatic nerve in the spared nerve injury model of neuropathic pain. Previous proteomic studies from spinal cord or DRG tissue found modifications of various proteins in neuropathic pain models (reviewed in [5]). A comparison of the results from different studies shows partly overlapping but mostly inconsistent protein modulations which might be due to the use of different nerve injury models, different animal strains or analysis of different neuronal tissues, and may also result from technical differences such as protein extraction protocols, coverage of the isoelectric focussing and molecular weight range, gel development and mass spectrometry detection. The Spared Nerve Injury model of neuropathic pain has not yet been investigated in terms of proteomics but was previously analysed for changes in mRNA expression in microarray studies [28]. In the present study we identified modifications of proteins involved in energy metabolism, maintenance of cellular structure and adherence, signal transduction and DNA binding. Some of these proteins were identified previously in proteomic studies with other nerve injury models (shown in Table S1), indicating that these proteins might contribute to a robust model-independent response to nerve injury and/or abundant expression and strong regulation of these proteins upon nerve injury, such as the mitochondrial protein prohibitin or the ubiquitin hydrolase L1. However, the bulk of the detected proteins were not found in previous proteomic analyses suggesting that they may be specifically regulated in the SNI model. A comparison between mRNA and protein levels of the respective proteins revealed that a number of regulations occur at the transcriptional level while several others appear to be translational or posttranslational modifications. Changes of protein stability and degradation may represent important immediate but also long lasting adaptive responses to the nerve injury. Because proteases determine longevity of proteins we focused on the protease inhibitor latexin as a candidate protein for further functional analysis. Recent reports showed that proteases of the metalloproteinase group [29] as well as some cathepsins [30] play important roles in the activation of glial cells in the spinal cord after nerve injury and the development of neuropathic pain. We found abundant latexin mRNA in dorsal and ventral horn neurons of the spinal cord and a dramatically diminished neuronal latexin expression on the side ipsilateral to the peripheral sciatic nerve lesion. Latexin is expressed in small-diameter peptidergic CGRP and substance P positive neurons in dorsal root ganglia [14] i.e. a proportion of nociceptive neurons. Previously, latexin knockout mice displayed an increased tail flick latency as compared to wild type animals upon noxious heat stimulation [15] suggesting that the here observed dramatic loss of latexin expression after nerve injury in spinal cord neurons may impact on the manifestation of neuropathic pain. Latexin is the only known endogenous carboxypeptidase A inhibitor in mammals [7] and in this regard it may be important to balance protein degradation in injured neurons [31]. We expected that the nerve injury evoked disappearance of latexin in spinal cord neurons should increase carboxypeptidase A activity and possibly enhance protein breakdown of carboxypeptidase substrate proteins or peptides. Among them are the endogenous opiod leu-enkephalin [32] and endothelin-1 [33] which may impact on the endogenous ability of nociceptive control [34], [35]. Increased cleavage particularly of endogenous opioid peptides might contribute to the development of neuropathic pain. The reciprocal relationship between latexin expression and carboxypeptidase A activity was confirmed in our study. Furthermore, we were able to prevent both the latexin disappearance and CPA enhancement with a rAAV2-mediated restoration of latexin expression specifically in neurons. Latexin restoration and inhibition of carboxypeptidase was associated with attenuated neuropathic cold pain-like behavior. We did not observe differences in heat pain sensitivity (not shown) which had been reported in latexin deficient mice [15]. Latexin knockout mice had shown prolonged paw withdrawal latency times upon acute heat stimulation in the Hot Plate test, indicating a reduced sensitivity to heat pain [15]. It is conceivable that CPA may have opposite effects in acute and nerve injury evoked nociception. However so far, exaggerated protease activity has been mainly observed in the context of chronic enhancement of inflammatory pain or nerve injury-evoked nociception. The interpretation of nociceptive behavior in latexin deficient mice is hampered by the general knockout approach because latexin deficiency may disturb neuronal development. Latexin expression in cortical neurons occurs early during embryogenesis at E11 and plays a role in the regional specification and morphogenesis of the forebrain [36]. In line with our observations in adult rodents sectioning of the sciatic nerve in P2 neonates also resulted in a dramatic reduction of latexin immunoreactivity in the spinal cord [14] indicating that this adaptation was fully functioning at birth. Prevention of the consequent CPA enhancement might be useful to attenuate nerve injury evoked nociceptive hypersensitivity, particularly cold allodynia. We do not know why carboxypeptidase inhibition was specifically important for cold allodynia but not mechanical hyperalgesia. It might be expected that latexin and carboxypeptidase are specifically important for cold-responsive nociceptive neurons to control protein cleavage. Latexin expression in the DRGs however, was not restricted to TRPA1 positive neurons. Nonetheless, subsets of specific neurons may be particularly sensitive to latexin-CPA dys-balances owing to cleavage of a CPA-subtype specific substrate [37]. So far, 6 CPA subtypes have been characterized which may be differentially expressed in subsets of neurons. From anatomical studies of the cortex it had been previously inferred that an area- and lamina-specific distribution of latexin-expressing neuronal subpopulations was important for the functional specialization of the cortical areas [38]. Such latexin-dependent specialization might also occur in the spinal cord. In summary, our results show that it is feasible to restore protein expression by AAV-mediated gene delivery in the spinal cord and thereby restore the balance between protease and endogenous inhibitor to reduce nerve injury evoked cold allodynia. Specific small molecule CPA inhibitors might be useful to reduce cold pain.

## Supporting Information

Table S1 Regulated proteins in the spinal cord 7 days after spared nerve injury.
(DOCX)
