# Supplementary material for: Nerve Injury Evoked Loss of Latexin Expression in Spinal Cord Neurons Contributes to the Development of Neuropathic Pain
Source: PLoS One. 2011 Apr 29;6(4):e19270. doi: 10.1371/journal.pone.0019270 (PMC3084808; doi:10.1371/journal.pone.0019270)
Supplement: Table S1 — Regulated proteins in the spinal cord 7 days after spared nerve injury. (DOCX) [file pone.0019270.s001.docx]

**Table S1**: Regulated proteins in the spinal cord 7 days after spared nerve injury.

| **Spot** | **Protein identified** | **T-test** | **Av. Ratio** | **MW calculated** | **pI calculated** | **MW observed** | **pI**  **observed** | **Mowse**  **Score** | **Masses matched** | **Masses**  **searched** | **%**  **Seq.Cov.** | **NCBI acc.No** | **Previous studies** |
| --- | --- | --- | --- | --- | --- | --- | --- | --- | --- | --- | --- | --- | --- |
| 1724 | Pyruvate dehydrogenase | 0.048 | -1.46 | 39.3 | 6.2 | 39 | 6.2 | 102 | 18 | 68 | 42 | 56090293 | Kang. 2006 |
| 1772 | Ubiquitin carboxy-terminal hydrolase L1 | 0.045 | -1.54 | 25.2 | 5.14 | 32 | 6.27 | 131 | 13 | 71 | 85 | 61098212 | Jimenez. 2005; Zhang. 2008; Huang. 2008 |
| 1773 | Protein-L-isoaspartate (D-aspartate) O-methyltransferase 1 | 0.015 | -1.53 | 24.7 | 7.14 | 32 | 9 | 76 | 8 | 53 | 58 | 56961640 |  |
| 1857 | Myelin basic protein | 0.013 | -1.84 | 14.2 | 11.75 | 29 | 8.99 | 77 | 8 | 55 | 57 | 4454311 |  |
| 1884 | Phosphoglycerate mutase 1 | 0.0099 | -1.99 | 28.7 | 6.19 | 28 | 8.4 | 103 | 14 | 76 | 45 | 16757984 |  |
| 1887 | Triosephosphate isomerase 1 (Tpi1) | 0.0048 | -1.89 | 27.2 | 7.07 | 28 | 6.87 | 275 | 23 | 79 | 93 | 38512111 | Jimenez. 2005 |
| 1895 | Ubiquitin carboxy-terminal hydrolase L1 | 0.0091 | -1.97 | 25.2 | 5.14 | 28 | 9.09 | 103 | 11 | 67 | 65 | 61098212 |  |
| 1911 | Nucleoside-diphosphate kinase 2 | 0.0056 | -2.21 | 17.4 | 6.92 | 28 | 8.39 | 119 | 11 | 37 | 57 | 55926145 |  |
| 1931 | Similar to cytochrome c oxidase. subunit vib polypeptide | 0.0056 | -2.37 | 18.2 | 8.9 | 27 | 8.55 | 79 | 8 | 50 | 59 | 62644353 |  |
| 1935 | Annexin A4 | 0.0028 | -2.02 | 36.2 | 5.42 | 36 | 5.42 | 86 | 13 | 70 | 46 | 55742832 | Huang. 2008 |
| 1937 | Latexin | 0.011 | -1.97 | 25.7 | 5.77 | 27 | 9.65 | 86 | 9 | 48 | 45 | 14269568 |  |
| 1940 | Glutathion-S-transferase. mu 5 | 0.0061 | -2.12 | 27.1 | 6.33 | 27 | 8.09 | 125 | 15 | 59 | 66 | 25282395 |  |
| 1944 | Triosephosphate isomerase 1 | 0.0085 | -2.22 | 27.2 | 7.07 | 27 | 9.16 | 82 | 9 | 52 | 36 | 38512111 |  |
| 1949 | Peptidylprolyl isomerase A | 0.0089 | -2.24 | 18.1 | 8.34 | 18 | 8.34 | 77 | 9 | 58 | 56 | 8394009 |  |
| 1960 | Voltage-dependent anion channel 2 | 0.0023 | -1.9 | 32.3 | 7.4 | 27 | 7.15 | 76 | 10 | 80 | 46 | 13786202 |  |
| 1965 | Phosphoglycerate mutase 1 | 0.0044 | -2.33 | 28.9 | 7.44 | 26 | 8.42 | 172 | 23 | 96 | 72 | 8248819 |  |
| 1970 | Triosephosphate isomerase 1 | 0.0018 | -2.15 | 27.2 | 7.07 | 26 | 5.95 | 266 | 22 | 71 | 93 | 38512111 |  |
| 1972 | Adenylate kinase 1 | 0.003 | -2.04 | 21.7 | 5.14 | 26 | 6.22 | 68 | 10 | 81 | 50 | 59808167 |  |
| 1973 | Peroxiredoxin 5. precursor | 0.029 | -1.57 | 22.5 | 7.66 | 26 | 9.78 | 115 | 12 | 83 | 63 | 51261175 |  |
| 1982 | Alpha-1-inhbitor III | 0.0047 | -2.22 | 31.6 | 5.9 | 26 | 9.19 | 52 | 3 | 23 | 41 | [19774135](http://www.matrixscience.com/cgi/protein_view.pl?file=../data/20050725/FsnpSnaS.dat&hit=1) |  |
| 1985 | ATP synthase, gamma subunit | 0.0052 | -1.89 | 33.1 | 9.06 | 26 | 7.26 | 45 | 7 | 52 | 17 | 39930503 |  |
| 1989 | NADH dehydrogenase (ubiquinone) Fe-S protein 3 | 0.002 | -2.1 | 30.4 | 7.07 | 26 | 7.69 | 99 | 12 | 79 | 31 | 20071222 |  |
| 1999 | Tpi1 protein | 0.011 | -2.08 | 27.2 | 7.07 | 25 | 8.96 | 111 | 11 | 53 | 46 | [38512111](http://www.matrixscience.com/cgi/protein_view.pl?file=../data/20050725/Fsnpmxua.dat&hit=1) |  |
| 2006 | Glutathione S-transferase. mu type 3 | 0.00072 | -2.07 | 25.8 | 6.84 | 25 | 5.9 | 192 | 23 | 76 | 71 | [13592152](http://www.matrixscience.com/cgi/protein_view.pl?file=../data/20050725/FsnpmxSt.dat&hit=1) |  |
| 2030 | Integrin linked kinase | 0.019 | -1.56 | 51.8 | 8.3 | 25 | 6.56 | 41 | 7 | 33 | 19 | 19173772 |  |
| 2089 | Prohibitin | 0.0021 | -2.12 | 29.9 | 5.57 | 21 | 8.26 | 82 | 9 | 52 | 41 | 13937353 | Zhang. 2008 |
| 2106 | Phosphoglycerate mutase type B subunit | 0.003 | -1.96 | 28.9 | 7.07 | 20 | 5.85 | 77 | 12 | 84 | 48 | 8248819 | Kang. 2006 |
| 2118 | Triosephosphate isomerase 1 | 3.30E-05 | -2.32 | 27.2 | 7.07 | 19 | 9.58 | 214 | 20 | 85 | 85 | [38512111](http://www.matrixscience.com/cgi/protein_view.pl?file=../data/20050725/Fsnpmxse.dat&hit=1) |  |
| 2121 | Psma2 protein | 0.019 | -1.41 | 26 | 6.92 | 19 | 7.45 | 62 | 7 | 60 | 41 | 8394063 |  |
| 2122 | Myelin basic protein | 1.80E-05 | -2.82 | 18.5 | 11.15 | 19 | 9.32 | 122 | 11 | 48 | 59 | [4454315](http://www.matrixscience.com/cgi/protein_view.pl?file=../data/20050725/FsnpmbuE.dat&hit=1) |  |
| 2123 | Myelin basic protein | 0.0035 | -1.52 | 14.2 | 11.15 | 14 | 11.15 | 121 | 10 | 42 | 60 | [4454311](http://www.matrixscience.com/cgi/protein_view.pl?file=../data/20050725/FsnpmecS.dat&hit=1) |  |
| 2125 | Major beta-hemoglobin | 5.10E-05 | -2.28 | 16.1 | 7.88 | 19 | 9.68 | 189 | 15 | 57 | 83 | [204570](http://www.matrixscience.com/cgi/protein_view.pl?file=../data/20050725/FsnpSnsm.dat&hit=1) |  |
| 2160 | Peroxiredoxin 6 | 0.00021 | -1.88 | 24.9 | 5.64 | 17 | 9.07 | 88 | 9 | 50 | 44 | 16758348 | Komori. 2007 |
| 2169 | Peroxiredoxin 6 | 0.00053 | -1.88 | 24.9 | 5.64 | 17 | 8.76 | 51 | 7 | 72 | 32 | 16758348 | Komori. 2007 |
| 2179 | Integrin linked kinase | 0.0013 | -1.76 | 51.8 | 8.3 | 17 | 7.65 | 41 | 7 | 34 | 22 | 19173772 |  |
| 2180 | Myelin basic protein | 0.0047 | -1.56 | 14.2 | 11.75 | 17 | 8.4 | 117 | 12 | 62 | 60 | [4454311](http://www.matrixscience.com/cgi/protein_view.pl?file=../data/20050725/Fsnpmect.dat&hit=1) |  |
| 2195 | Triosephosphate isomerase 1 | 0.0036 | -1.9 | 27.2 | 7.07 | 16 | 7.07 | 196 | 19 | 82 | 79 | [38512111](http://www.matrixscience.com/cgi/protein_view.pl?file=../data/20050725/FsnpmiEw.dat&hit=1) |  |
| 2198 | Peroxiredoxin 6 | 0.00014 | -1.8 | 24.9 | 5.64 | 16 | 9.59 | 123 | 13 | 58 | 52 | 16758348 |  |
| 2215 | Nucleoside-diphosphate kinase 2 | 0.00025 | -1.65 | 17.4 | 6.92 | 15 | 8.66 | 60 | 6 | 35 | 43 | [55926145](http://www.matrixscience.com/cgi/protein_view.pl?file=../data/20050725/FsnpmbuT.dat&hit=1) |  |
| 2224 | Myelin basic protein | 0.00023 | -1.45 | 14.2 | 11.75 | 15 | 8.91 | 127 | 13 | 52 | 60 | [4454311](http://www.matrixscience.com/cgi/protein_view.pl?file=../data/20050725/Fsnpmeaa.dat&hit=1) |  |
| 2248 | Cyt P-450 (AA at 72) | 2.50E-09 | 5.16 | 12.5 | 5.8 | 13 | 11.92 | 56 | 5 | 47 | 50 | [554434](http://www.matrixscience.com/cgi/protein_view.pl?file=../data/20050725/FsnpSnHT.dat&hit=1) |  |
